# Breast Mass Classification Using Diverse Contextual Information and Convolutional Neural Network

**DOI:** 10.3390/bios11110419

**Published:** 2021-10-26

**Authors:** Mariam Busaleh, Muhammad Hussain, Hatim A. Aboalsamh, Fazal-e- Amin

**Affiliations:** 1 Department of Computer Science, CCIS, King Saud University, Riyadh 11451, Saudi Arabia; mhussain@ksu.edu.sa (M.H.); hatim@ksu.edu.sa (H.A.A.); 2 Department of Software Engineering, CCIS, King Saud University, Riyadh 11543, Saudi Arabia; famin@ksu.edu.sa

**Keywords:** breast mass classification, mammography, transfer learning, BIRADS, convolutional neural network (CNN), ensemble classifier

## Abstract

Masses are one of the early signs of breast cancer, and the survival rate of women suffering from breast cancer can be improved if masses can be correctly identified as benign or malignant. However, their classification is challenging due to the similarity in texture patterns of both types of mass. The existing methods for this problem have low sensitivity and specificity. Based on the hypothesis that diverse contextual information of a mass region forms a strong indicator for discriminating benign and malignant masses and the idea of the ensemble classifier, we introduce a computer-aided system for this problem. The system uses multiple regions of interest (ROIs) encompassing a mass region for modeling diverse contextual information, a single ResNet-50 model (or its density-specific modification) as a backbone for local decisions, and stacking with SVM as a base model to predict the final decision. A data augmentation technique is introduced for fine-tuning the backbone model. The system was thoroughly evaluated on the benchmark CBIS-DDSM dataset using its provided data split protocol, and it achieved a sensitivity of 98.48% and a specificity of 92.31%. Furthermore, it was found that the system gives higher performance if it is trained and tested using the data from a specific breast density BI-RADS class. The system does not need to fine-tune/train multiple CNN models; it introduces diverse contextual information by multiple ROIs. The comparison shows that the method outperforms the state-of-the-art methods for classifying mass regions into benign and malignant. It will help radiologists reduce their burden and enhance their sensitivity in the prediction of malignant masses.

## 1. Introduction

Breast cancer is one of the deadliest and most common cancers among women. According to a World Health Organization (WHO) report, breast cancer accounts for 2.26 million of all diagnosed cancers and 685,000 cancer-related deaths worldwide [1]. Mammography is the dominant screening procedure for the early diagnosis of this cancer; and micro-calcifications and masses are its early signs [2,3]. As the mammogram images have low contrast, discrimination of benign and malignant masses is challenging [4]. A CAD system first identifies the mass regions and then classifies them into benign and malignant. We focused on the second problem of CAD, i.e., classifying the mass regions into benign or malignant, which is a very difficult problem to resolve [5]. Recently, medical imaging researchers used innovative deep learning methods to overcome this problem, but their performance is low and may not be acceptable for clinical use [6,7,8]. An ensemble classifier can be used for better performance because an ensemble classifier strategy achieves a more promising performance than using a single classifier. However, adopting this method for deep learning-based classifiers is costly in memory and computing complexity since training and storing many CNN models is costly. Based on the hypothesis that diverse contextual information helps better discriminate benign and malignant masses and the idea of an ensemble classifier, we introduce a method for automatically classifying mass regions into benign and malignant. It is computationally efficient, effective, and requires less memory. Rather than learning different CNN models, it learns only one model and adds diversity in strategic decisions using different variations of the same unseen pattern. We validated the system using the Curated Breast Imaging Subset of the Digital Database for Screening Mammography (CBIS-DDSM) dataset and the INbreast dataset as challenging datasets. The main contributions of this study are as follows:

- We proposed a system for the classification of masses into benign and malignant using contextual information. It is based on the idea of an ensemble classifier and uses ResNet-50 as the backbone CNN model. It introduces diversity in decision-making using contextual information with multiple ROIs, not using multiple CNN models.
- For the system, we employed two schemes to extract multiple ROIs from a mass region for modeling diverse contextual information. For fusing the diverse contextual information from different ROIs, we used different fusing techniques to find the best one. Stacking gives the best results.
- We employed the ResNet-50 model as a backbone model. To examine the effect of breast density in the discrimination of benign and malignant masses, we introduced density-specific modifications of ResNet-50 based on the idea of fusing local and global features, knowing that when the density-specific model is used, the breast density must be known. The density-specific models result in better performance than ResNet-50. Finally, the impact of different BI-RADS types on the classification was evaluated.
- The mass regions of each type are not enough to fine-tune even a pre-trained model. We introduced a data augmentation approach for fine-tuning the pre-trained models.

The rest of the paper is structured as follows. Section 1 presents a review of related literature. Section 2 describes the proposed system for the discrimination of benign and malignant masses, fine-tuning of backbone CNN models, data augmentation, and evaluation protocols. Experimental results, as well as their interpretation and discussion, are presented in Section 3 and Section 4. Finally, the conclusions and future work are summarized in Section 5.

### Related Work

The problem of classifying the mass region into benign and malignant has attracted the attention of many researchers, and many methods have been proposed based on hand-engineered feature-based methods and deep learning-based methods. These methods have been evaluated using the benchmark datasets such as Digital Database for Screening Mammography (DDSM), Curated Breast Imaging Subset of DDSM Digital Database for Screening Mammography (CBIS-DDSM), Mammographic Image Analysis Society (mini-MIAS), and INBreast. Among these datasets, CBIS-DDSM is the most challenging and provides a well-defined evaluation split protocol. In the following paragraphs, a brief review of the published research works is presented, which strictly followed the evaluation protocol of CBIS-DDSM.

Khan et al. [8] proposed a three-stage CAD system based on Multi-View Feature Fusion (MVFF), which takes four mammography views as input, extracts CNN features from four views, fuses them using concatenation, and finally classifies them into normal/abnormal, or mass/calcification, and benign/malignant. Although this method uses very rich information as input, its performance (sensitivity of 81.82%, and specificity of 72.02%) for benign and malignant classification is very poor. This is because each view is rescaled to 128 × 128, which causes it to throw away a lot of discriminative information. Li et al. [9] proposed a Dual Path Conditional Residual Network (DUALCORENET), where the first path learns the texture features, and the second path learns the input-mask correlation. Finally, features of the dual paths are concatenated for mass classification. It gave an AUC of 85%. Tsochatzidis et al. [10] compared state-of-the-art CNN models to identify the pathology of an ROI. They employed two mechanisms to train a CNN model: training from scratch and fine-tuning a pre-trained model. They showed that fine-tuning gives better results, and among different CNN models, ResNet-50 achieved the best performance (80.40% of AUC and 74.90% of ACC). Duggento et al. [11] developed an ad hoc random initialization deep neural network to classify mass regions into benign and malignant without using the pre-trained public models commonly used for transfer learning. They explored a total of 260 model architectures in a train-validation-test split to suggest a model selection criterion that may emphasize minimizing false negatives while still preserving reasonable accuracy. This method achieved an accuracy of 71.19%, sensitivity of 84.40%, and specificity of 62.44%. Shu et al. [12] designed an end-to-end CNN architecture inspired by DenseNet to classify the entire mammographic image. Two different pooling structures have been proposed rather than a common pooling method to pool the feature map. Alhakeem, and Jang [13] proposed a texture-based approach that extracts features using an integrated form of a matrix-based local binary pattern (M-LBP) and a matrix-based histogram of oriented gradients (M-HOG) descriptors based on global matrix projection and classifies masses using an LSE classifier. M-LBP-HOG and LBP-HOG achieved accuracies of 64.35% and 62.07%, respectively. Chougrad et al. [14] developed a CAD system to predict the mass lesions into benign or malignant. They used deep learning with transfer learning in various convolution neural network models from different public datasets. The results of the ResNet-50 on the INbreast test set overall 5-fold cross-validations achieved 95.50% accuracy and 0.97 AUC. Al-antari et al. [15] proposed a deep learning-based CAD system for detecting, segmenting, and classifying masses in INbreast datasets. The results of classification verified its performance through four-cross validation utilizing AlexNet (95.64% of ACC and 94.78% of AUC). Shen et al. [16] developed an end-to-end residual-aided classification U-Net model (ResCU-Net) for mass segmentation and classification of mammograms simultaneously. This method achieved an accuracy of 94.16%, sensitivity of 93.11%, and specificity of 95.02% verified through three-fold cross-validation. Ghada et al. [17] proposed YOLO based CAD system to detect masses that may exist in the mammogram. They used Specific CNN models (ResNet and Inception V3) to classifies masses into benign/malignant. InceptionV3 achieves the best classification results, with an overall classification accuracy of 95%. Lou et al. [18] designed an end-to-end multi-level global-guided branch-attention network (MGBN) for mass classification into benign/malignant. The MGBN based on ResNet-50 provides the highest AUC value for mass classification, with 0.8375 for the DDSM database and 0.9311 for the INbreast database, respectively.

The above overview of the state-of-the-art methods reveals that the classification of a mass region into benign and malignant needs more research. All the methods discussed above using a single classifier rather than an ensemble classifier. Ensemble classifier plays a key role in improving the prediction accuracy of a classification model [19]. Furthermore, using various data augmentation techniques to increase the amount of training data available to the CNN allow it to learn more mass characteristics. Previous studies have based their criteria for training data augmentation on commonly used transformations such as flipped, rotation, shear, zoom, angle, and so on. Our specific augmented training dataset includes the common transformation along with contextual information, which has a significant impact on performance

Shen et al. [20] addressed the false positive reduction problem to identify an ROI into a mass or normal and employed multi-context information using multi-scale ROIs around a suspicious region. They have shown that multi-context information results in significant improvement. We also imply multi-context information to discriminate benign and malignant masses, but our approach is different.

## 2. Proposed Method

We introduce a novel ensemble-based system for the classification of mammogram masses into benign and malignant. A common boosting approach in the design of an ensemble is to use a set of weak diverse learners; to induce the diversity in the learners, the same model is trained with different samples of the data, usually created with bootstrap sampling. The class of an unknown pattern is predicted by passing it to each learner and fusing their predictions. This approach results in better classification performance than using a single classifier [21,22,23,24,25,26]. However, to adopt this approach for deep learning-based classifiers is very expensive from the point of view of computational and storage space complexity because to train many CNN models and store them is very expensive. To design an ensemble classifier based on CNN, we introduce a different approach, which is computationally very efficient and needs less storage space. Instead of learning weak diverse CNN models, we learn only one model and introduce diversity in decision making by using diverse versions of the same unknown pattern. Another problem is that the CNN model is suspected of adversarial attack [27]; the deep model may mis-classify the mass when using only one ROI. The surrounding context of a mass object in a mammogram image forms an important clue for discriminating masses [20,28]. Observing varying contexts around the same mass object introduces diversity and can better help us understand the nature of a mass.

**Figure 1:**
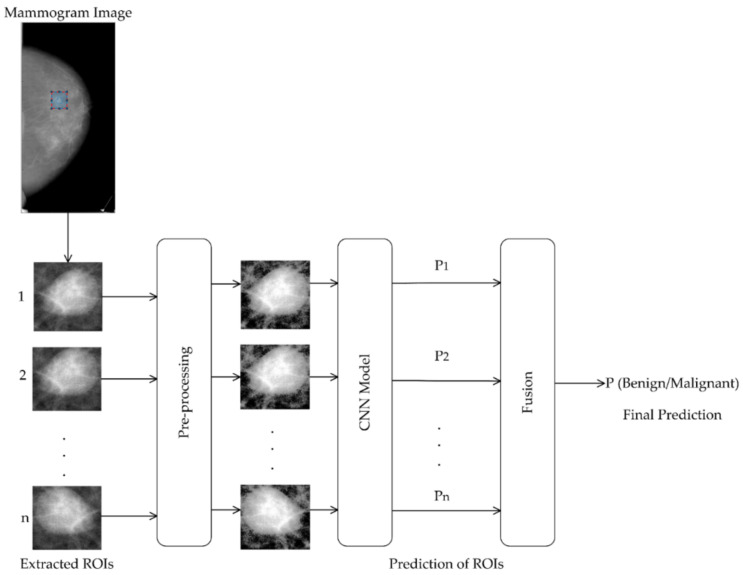
Proposed mammogram classification system.

Based on this observation, we extract ROIs of the same mass region with different contexts and pass them to the same CNN model; the predictions of these ROIs are then fused to take the final decision whether the mass is benign or malignant. The design of such an ensemble CNN model is shown in Figure 1. It overcomes the two issues stated above. The development of this classifier involves three key design decisions: (i) which approach is suitable for extracting ROIs around a mass with varying contexts, (ii) which CNN model is suitable for this ensemble, and (iii) which fusion technique yields the best prediction results. In the following subsections, we give the detail about each of these design decisions.

### 2.1. Modeling the Context Information

We introduce two approaches to crop multiple ROIs with diverse contexts from an unknown mass region. It is assumed that the mass region has already been segmented, and the problem is to classify it as benign or malignant. For our experiments, we used the annotation provided by the CBIS-DDSM database to obtain the segmented mass regions. To generate ROIs with different contexts from a mass region, the mass region with varying amounts of surrounding tissues are cropped. Furthermore, we resize each ROI to (224 × 224) pixels to support the input size of the pre-trained CNN models such as ResNet-50.

#### 2.1.1. Scale-Based Multi-Context Regions of Interest (ROIs) Extraction

**Figure 2:**
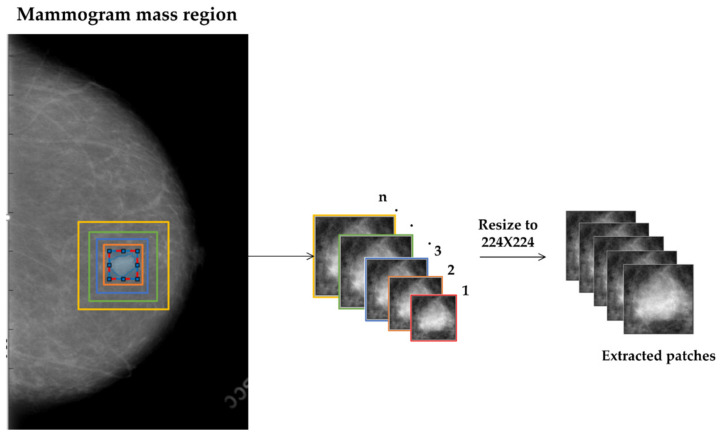
The regions of interest (ROIs) with different contextual information, modeled using scale-based technique.

A multi-scale cropping mechanism is used to extract the ROIs with different scales around a mass region. We expand the bounding box of the mass region by a fixed ratio 5:5 or 10:10 to crop n ROIs that contain a variety of contextual information and allow a CNN model to pay attention to this information surrounding the mass for decision making [20,28]. Figure 2 demonstrates this approach. As the ROIs are resized to the fixed size of 224 × 224, the contextual information increases with increasing the scale of cropping.

#### 2.1.2. Translation-Based Multi-Context ROIs Extraction

**Figure 3:**
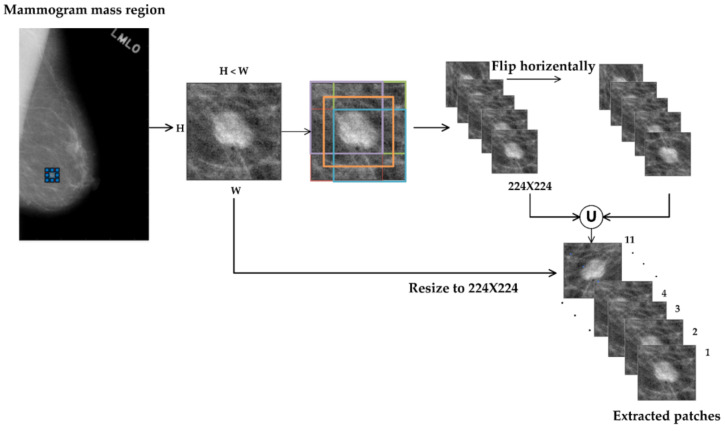
The ROIs with different contextual information, modeled using translation-based technique.

In general, a mass region does not have the same dimensions, i.e., the height and width are not the same. In this case, the anisotropic transformation, used to resize an ROI to a fixed size of 224 × 224, introduces distortion in the ROI and distorts the texture patterns. To avoid this problem and extract ROIs with different contexts, we employ a technique inspired by the method adopted in [29,30]. First, the mass region is rescaled isotropically so that its smaller side is transformed to the base scale of 256 pixels. This means that we do scaling so that the aspect ratio is preserved and there is no distortion or deformation. Then, we crop 4 ROIs, each of size 224 × 224, from the four corners (top-left, top-right, bottom-left, and bottom-right) of the rescaled mass region and one from its center to have 5 ROIs with different contexts and of a fixed size of 224 × 224. Finally, we flip them horizontally; in this way, we obtain 11 ROIs with different contexts, allowing the CNN model to make different decisions for different ROIs. Unlike the previous approach, in this case, the diversity in the contextual detail is introduced without scaling the mass object into different scales. Figure 3 illustrates this process.

### 2.2. Preprocessing

**Figure 4:**
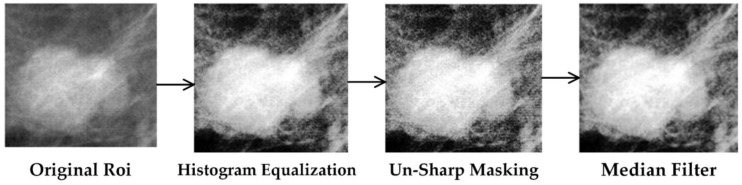
Sample of preprocessed mammogram ROI.

The contrast of soft tissues in mammogram images of CBIS-DDSM is low; it must be enhanced to distinguish mammographic lesions with low visibility and contrast. We apply histogram equalization followed by un-sharp masking with a sharpening strength of 0.8 to improve the contrast of ROIs. Finally, a median filter with kernel size 3 × 3 is used to reduce the noise [31]. Figure 4 shows an example of enhanced ROIs. Also, please note that we did not use any preprocessing for INBreast, because the images already have good contrast.

### 2.3. Backbone Convolutional Neural Network (CNN) Model

**Figure 5:**
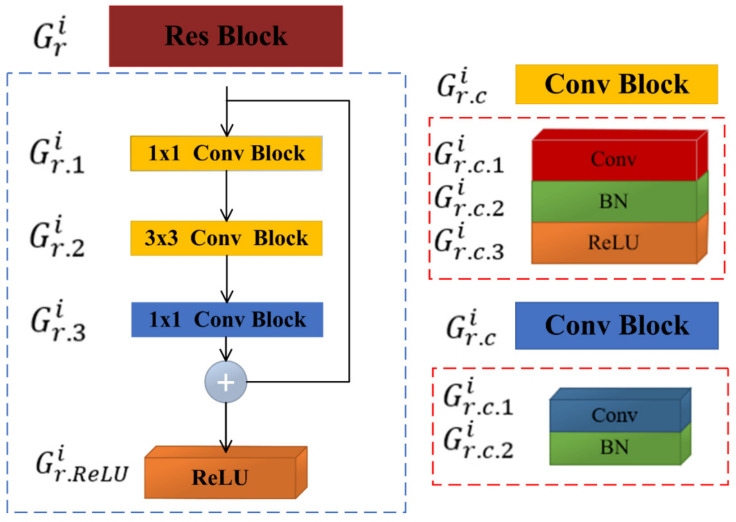
Bottleneck building block.

In this study, motivated by the related work, we adopted ResNet-50 as a backbone model to classify mammogram mass ROIs as benign or malignant [8,10] and also modified it to adapt it to breast density. Its architecture is based on residual theory, which allows increasing the depth of a CNN model without suffering from degradation problems [32]. Deeper CNN models learn discriminative features and boost the classification performance. The ResNet-50 model proposed by He et al. [32] serves as the basis for our design. This model consists of five groups $G^{i},\;i=1,2,3,4,5$ of residual blocks. Let $G_{r}^{i}$ denote the $r^{th}$ residual block of $i^{th}$ group, and $G_{r.c.l}^{i}$ represent the $l^{th}$ layer of $c^{th}$ convolutional block of $r^{th}$ residual block of $i^{th}$ group, and $G_{r.ReLU}^{i}$ stands for the ReLU layer of the $r^{th}$ residual block of $i^{th}$ group, as shown in Figure 5. Table 1 gives an overview of the architecture of ResNet-50; it is based on bottleneck design, and the basic building block is shown in Figure 5.

### 2.4. Fine-Tuned ResNet-50

As the first choice for the backbone model, we employed the original ResNet-50 model pre-trained on ImageNet [33] with transfer learning. We replaced the last classification layer of ResNet-50 with a new FC layer with two neurons because there are two classes, benign and malignant, and fine-tuned it, using training data consisting of all density types.

### 2.5. Density Specific Modification of ResNet-50–DResNet-50

We modified the ResNet-50 model to develop a breast density-specific mass classification system. Fusing both local (low-level) and global (high-level, semantics) features, a CNN model is capable of learning more discriminative information. Specifically, the global features pay more attention to the semantics of masses, while the local features capture mass-specific-fine details [34,35,36]. Inspired by the fully convolutional network (FCN) [37], we fused local and global features by adding projection shortcuts, which provide access to the activations of previous layers, making it possible to reuse low-level features [38] to improve network performance. We explored four different settings for designing the density-specific deep convolutional neural networks; we observed that the filters in the branches concentrate on particular low-level features and help boost the extraction of features specific to different breast densities [39]. Moreover, the fusion between global average and global max pooling activations contributes to the learning of mass and texture-level features [40]. Keeping in view these observations, we constructed the density-specific model for each density type.

#### 2.5.1. DIResNet-50 for BI-RADS I

**Figure 6:**
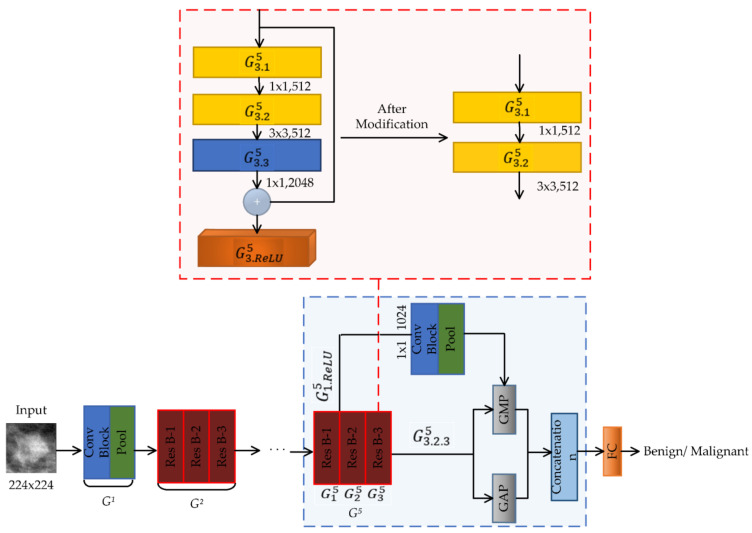
DIResNet-50 model.

To adapt the model for the density class BI-RADS I, we made modifications, keeping in view the motivations described above, and after trying different options, we called this DIResNet-50. First, we removed the Conv Block $G_{3.3}^{5}$, the ReLU layer $\;G_{3.ReLU}^{5}$ and the shortcut of $G_{3}^{5}$ (as shown in Figure 6) so that the output feature map of $G_{3}^{5}$ consisted of 512 channels. Then, we added a global average pool (GAP) layer in parallel to global max pool (GMP) after the Conv Block $G_{3.2}^{5}$, and a concatenation layer, as shown in Figure 6. After that, we added a projection shortcut from the ReLU layer $G_{1.ReLU}^{5}$ of $G_{1}^{5}$ to the concatenation layer; it consisted of a 1 × 1 Conv Block with 1024 filters followed by a 7 × 7 max-pooling layer, as shown in Figure 6. The modification was done so that the number of channels in the output feature map of the concatenation layer was 2048, which was the original number of channels. Finally, a fully connected layer (FC) with two neurons was introduced after the concatenation layer.

#### 2.5.2. DIIResNet-50 for BI-RADS II

**Figure 7:**
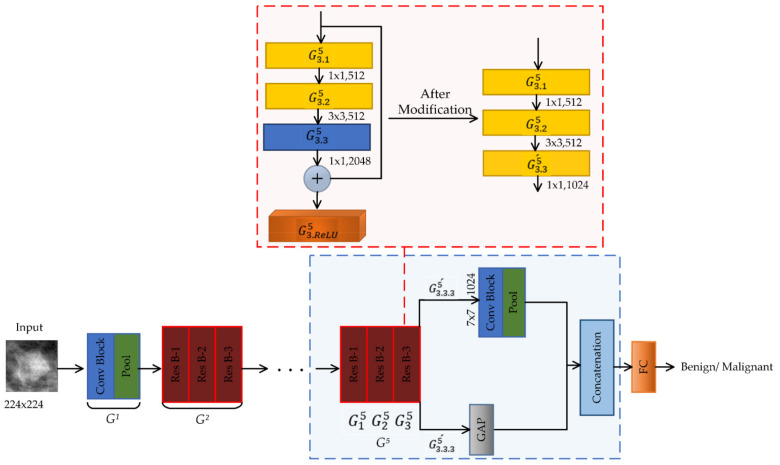
DIIResNet-50 model.

The model DIIResNet-50 is adapted for the density class BI-RADS II. First, we replace the Conv Block $G_{3.3}^{5}$ consisting of 2048 filters with the Conv Block $\overset{´}{G_{3.3}^{5}}$ having 1024 filters and remove the shortcut of $G_{3}^{5}$ (as shown in Figure 7). Then, we add a projection block consisting of a 1 × 1 Conv Block with 1024 filters and a 7 × 7 max pool layer after $\overset{´}{G_{3.3}^{5}}$ and parallel to the GAP layer. Afterward, to concatenate the activations of GAP and the projection block, we add a concatenation layer. Finally, an FC layer with two neurons is incorporated, as shown in Figure 7. The modifications are done so that the number of channels in the output feature map of the concatenation layer is 2048, which is the original number of channels.

#### 2.5.3. DIIIResNet-50 for BI-RADS III

**Figure 8:**
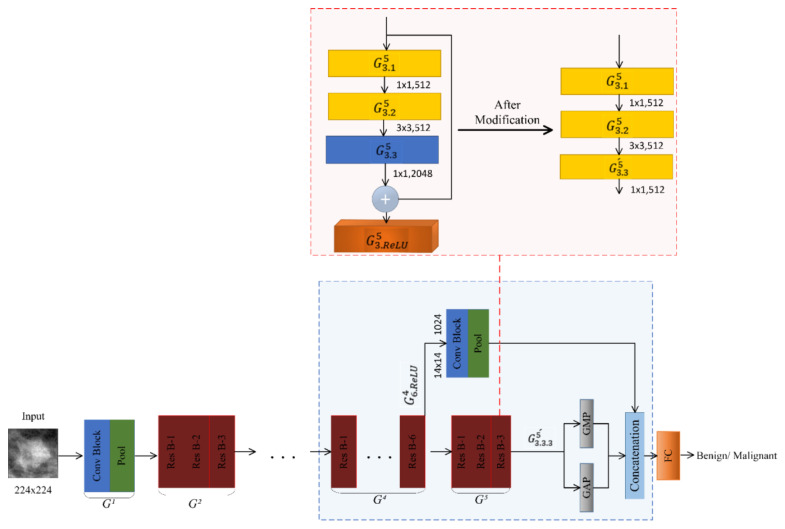
DIIIResNet-50 model.

To adapt the model for the density class BI-RADS III, we made modifications and call it DIIIResNet-50. First, we replace the Conv Block $G_{3.3}^{5}$ having 2048 filters with the Conv Block $\overset{´}{G_{3.3}^{5}}$ consisting of 512 filters and removed the shortcut of $G_{3}^{5}$. Then, we added a GMP layer in parallel to the GAP after the Conv Block $\overset{´}{G_{3.3}^{5}}$, and a concatenation layer, as shown in Figure 8. After that, we add a projection shortcut from the ReLU layer $G_{6.ReLU}^{4}$ of $G_{6}^{4}$ to the concatenation layer; it consists of a 1 × 1 Conv Block with 1024 filters followed by a 14 × 14 max-pooling layer, as shown in Figure 8. The modification is done so that the number of channels in the output feature map of the concatenation layer is 2048, which is the original number of channels. Finally, a FC with two neurons is introduced after the concatenation layer.

#### 2.5.4. DIVResNet-50 for BI-RADS IV

**Figure 9:**
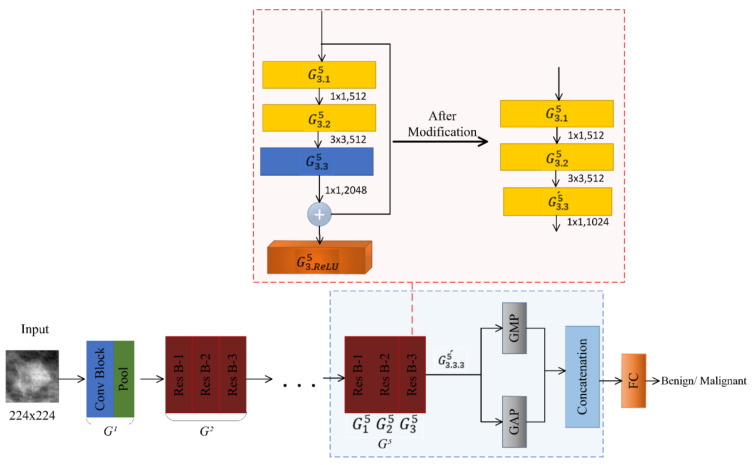
DIVResNet-50 model.

To adapt the model for the density class BI-RADS IV, we made modifications to ResNet-50 and call it DIVResNet-50. First, we replaced the Conv Block $G_{3.3}^{5}$ consisting of 2048 filters with the Conv Block $\overset{´}{G_{3.3}^{5}}$ having 1024 filters and removed the shortcut of $G_{3}^{5}$ (as shown in Figure 9). Then, we added a GMP layer in parallel to GAP after the ReLU layer $\;G_{3.3.3}^{5}$ of $G_{3}^{5}$, and a concatenation layer, as shown in Figure 9. The modification was done so that the number of channels in the output feature map of the concatenation layer was 2048, which was the original number of channels. Finally, a FC with two neurons was introduced after the concatenation layer.

### 2.6. Fusion Techniques

The next important task in the system was to fuse the predictions of multiple ROIs corresponding to a mass region. We adapt some well-known techniques.

#### 2.6.1. Majority of the Decisions

In this fusion technique, the class labels of multiple ROIs that correspond to a mass region are calculated using a backbone CNN model, and the predicted label (benign/malignant) of the mass region is the one that has maximum votes. To overcome the issue of a tie, we generate an odd number of ROIs. Let there are n ROIs$\;\left\{P_{1},\;P_{2},\;\ldots .,P_{n}\right\}$, extracted from a mass region. Their predicted labels from model M are $\left\{l_{1},\;l_{2},\;\ldots ,\;l_{n}\right\}$, the predicted label l of the mass region is computed using majority vote as follows [41]:

$$l=\mathrm{majority}\;\mathrm{of}\;\left\{l_{1},\;l_{2},\;\ldots ,\;l_{n}\right\}$$

#### 2.6.2. Soft Voting

In this technique, the predicted probabilities of each class are averaged over all ROIs, and the final predicted class of the mass region is the one for which the average predicted probability is larger. To be precise, let the predicted probabilities of n ROIs with the model M be $\left\{\left(p_{1}^{1},p_{2}^{1}\right),\left(p_{1}^{2},p_{2}^{2}\right),\;\left(p_{1}^{3},p_{2}^{3}\right)\ldots ,(p_{1}^{n},p_{2}^{n})\right\}$, and $p_{1}=\frac{\sum_{i=1}^{n}\left(p_{1}^{i}\right)}{n}\;$, $p_{2}=\frac{\sum_{i=1}^{n}(p_{2}^{i})}{n}$, then, the predicted label l of the region is computed as follows [41]:

$$l=\left\{\begin{array}{c} 1\;\left(benign\right)\;if\;p_{1}>p_{2}\;\\ 2\left(malig\right)\;otherwise \end{array}\right.$$

#### 2.6.3. Max Voting

In this technique, the maximum of predicted probabilities of each class are calculated over all ROIs, and the final predicted class of a mass region is the one for which the maximum predicted probability is greater. To be precise, let the predicted probabilities of n ROIs with the model M be $\left\{\left(p_{1}^{1},p_{2}^{1}\right),\left(p_{1}^{2},p_{2}^{2}\right),\;\left(p_{1}^{3},p_{2}^{3}\right)\ldots ,p_{1}^{n},p_{2}^{n})\right\}$, and $p_{1}=max(p_{1}^{1},p_{1}^{2},p_{1}^{3},\ldots ,p_{1}^{n}$); $p_{2}=max(p_{2}^{1},p_{2}^{2},p_{2}^{3},\ldots ,p_{2}^{n}$), then, the predicted label l of the ROI is computed as follows [41]:

$$l=\left\{\begin{array}{c} 1\;\left(benign\right)\;if\;p_{1}>p_{2}\;\\ 2\left(malig\right)\;otherwise \end{array}\right.$$

#### 2.6.4. Stacking

**Figure 10:**
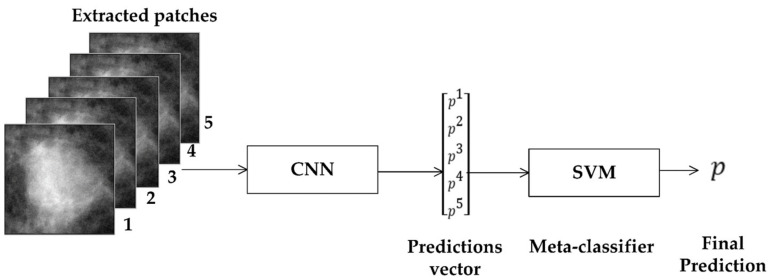
Stacked generalization method.

All the techniques discussed above make final decisions based on the predictions/labels only and do not consider the weights of ROIs. One technique that takes into account the weights of ROIs is stacking [42]. In this technique, the predictions of the ROIs are calculated using a backbone CNN model and stacked into a vector, which is passed to a classifier for predicting the label of the region, as shown in Figure 10. We can use any classifier since it is a two-class problem; we used SVM with different kernels. The design of SVM is based on a large margin theory and gives very good results in many binary classification problems [43,44,45,46]. The SVM model is trained using the predictions of the training and the validation datasets.

### 2.7. Training of CNN Models

#### 2.7.1. Datasets

To demonstrate the effectiveness and robustness of the proposed system, we used two public benchmark mammographic datasets:

1. CBIS-DDSM [47]. The Curated Breast Imaging Subset of DDSM (CBIS-DDSM) is a challenging dataset that contains digitized film images of 753 calcifications and 891 masses converted to Digital Imaging and Communications in Medicine (DICOM) format. This dataset has the updated annotations of mass regions on mediolateral oblique (MLO) and bilateral craniocaudal (CC) views. The database size and ground truth verification make the DDSM a useful tool in developing and testing support systems without any bias. Using the annotations, we extracted benign and malignant mass regions. We only considered the mass abnormality with breast density. For evaluation, we used the protocol provided for this dataset; the dataset is separated into train and test datasets, as shown in Table 2. In the sequel, D_T_, D1_T_, D2_T_, D3_T_, D4_T_ stand for the complete training data set (D1_T_∪D2_T_∪D3_T_∪D4_T_), and the training datasets for BI-RADS.I, BI-RADS.II, BI-RADS.III, BI-RADS.IV, respectively. Similarly, D_Ts_, D1_Ts_, D2_Ts_, D3_Ts_, D4_Ts_ represent the complete test data set (D1_Ts_∪D2_Ts_∪D3_Ts_∪D4_Ts_), and the test datasets for BI-RADS.I, BI-RADS.II, BI-RADS.III, BI-RADS.IV, respectively.
2. INbreast [48]. It is the largest public dataset that contains 410 full-field digital mammographic (FFDM) images provided in Digital Imaging and Communications in Medicine (DICOM) format. Each case consists of mediolateral oblique (MLO) and bilateral craniocaudal (CC) views. According to the database size and ground truth verification, the INbreast provides useful data for building and testing support systems without bias. According to the annotation, the statistics of this dataset are given in Table 3.

#### 2.7.2. Data Augmentation

**Figure 11:**
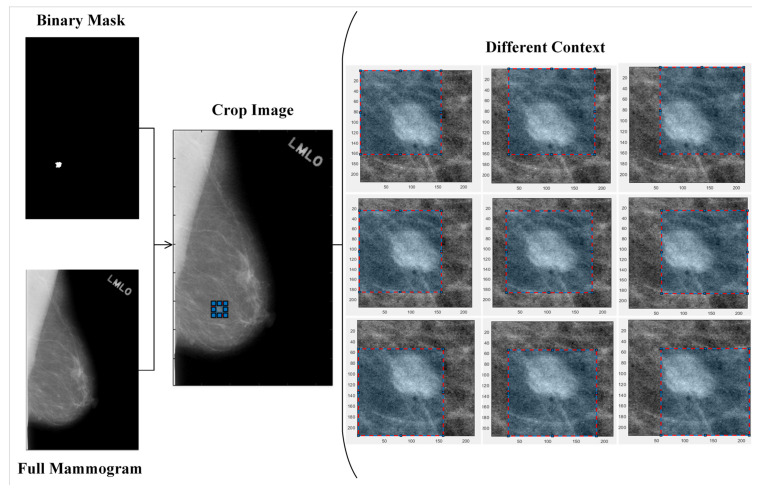
Extracted ROI with different contexts.

**Figure 12:**
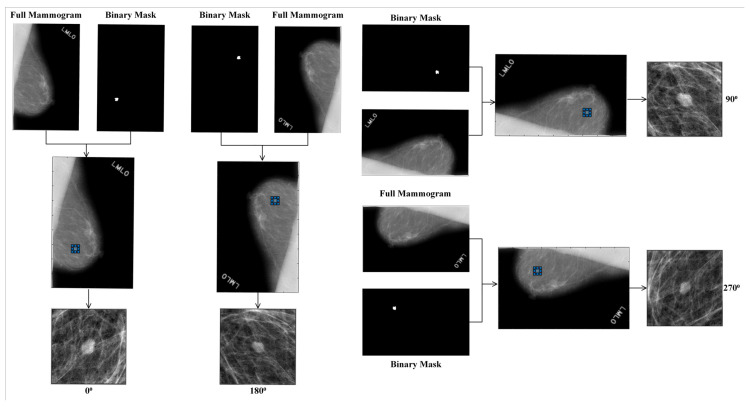
Extracted ROI with different orientations.

**Figure 13:**
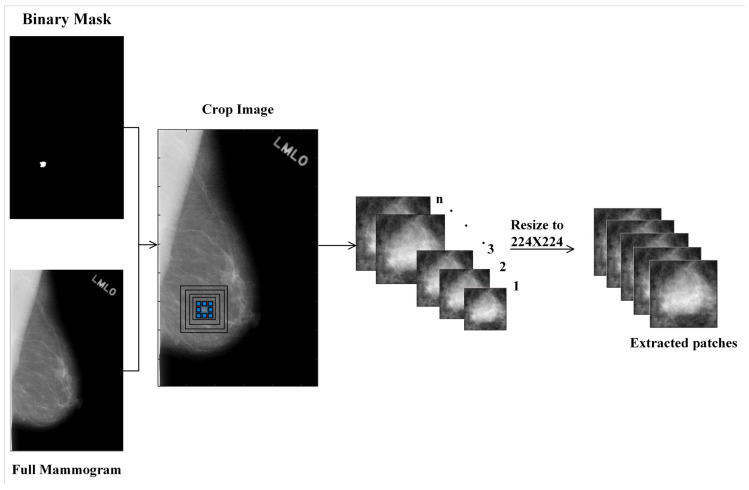
Extracted ROI with different scales.

Training a CNN model on a large number of training examples usually works well and provides high-performance values. However, Table 2 and Table 3 indicate that the number of training examples is small, so data augmentation is essential [49]. Also, the augmentation helps overcome the data imbalance problem. To simulate a large number of mass regions, we create multi-context, multi-orientation, and multi-scale ROIs from a mass region, as shown in Figure 11, Figure 12, Figure 13. A window of fixed size slides with 10 pixels stride to extract multi-context ROIs from a mass region so that the whole mass is inscribed in the window. For extracting multi-orientation ROIs, a mammogram image is rotated clockwise through θ, where θ∈{0°,90°,180°, 270°}, and ROIs are extracted and flipped. For extracting multi-scale ROIs, the bounding box of the mass region expanded by a fixed ratio of 5:5 or 10:10. Finally, the extracted ROIs are resized to (224 × 224).

#### 2.7.3. Fine-Tuning the Backbone Models

After modifications in the base pre-trained ResNet-50, each backbone CNN model is fine-tuned using the augmented training data. We fine-tuned the models with stochastic gradient descent with a momentum of 0.9 and a learning rate of 1 × 10^−4^. The mini-batch size was set to 64.

## 3. Evaluation Protocol

For the evaluation of the proposed system and fair comparison with the state-of-art methods:

- We used the evaluation protocol provided for CBIS-DDSM. The training set was used to fine-tune the backbone models; it was divided into training and validation sets with a ratio of 90:10. The new training set was utilized to fit the model independently, and the validation set was employed to control the training process. After completing a model’s training, its performance was evaluated on the test set of CBIS-DDSM without bias.
- For INbreast the cases are randomly divided into 80% for training,10% for validation, and 10% for testing, which allows us to run five-fold cross-validation. Cross-validation provides a less biased estimate of the model’s ability for unseen data.

The performance of the system was evaluated using the following well-known evaluation metrics [50,51]:

$$\begin{array}{c} \mathrm{Sensitivity}\;\left(\mathrm{Sen}.\;\right)=\frac{TP}{TP+FN}\;\mathrm{Accuracy}\;\left(\mathrm{Acc}.\;\right)=\frac{TP+TN}{TP+FN+TN+FP}\\ \mathrm{Specificity}\;\left(\mathrm{Spe}.\;\right)=\frac{TN}{TN+FP}\;F1\;\mathrm{Score}=2\times \frac{\mathrm{Sensitivity}\times \mathrm{Specificity}\;}{\mathrm{Sensitivity}+\mathrm{Specificity}}\\ \mathrm{Kappa}=\frac{\left(P_{o}-P_{e}\right)}{\left(1-P_{e}\right)}\\ \;P_{e}=\frac{\left(TP+FN\right)\times \left(TP+FP\right)+\left(FP+TN\right)\times \left(FN+TN\right)}{{\left(TP+TN+FP+FN\right)}^{2}}\;;\;P_{o}=\frac{TP+TN}{TP+TN+FP+FN} \end{array}$$

In addition, the area under the receiver operating characteristics curve (AUC) is also used to measure the performance.

The system was implemented, and all experiments were performed in MATLAB R2020a with a deep learning toolbox on an ASUS desktop with Intel Core i7-6800K CPU@3.4 32 GB RAM and GeForce RTX 2080Ti 12GB.

## 4. Result

This section presents the experimental results with different options for the backbone model, the scheme for modeling diverse contextual information, and the fusion technique.

### 4.1. Why ResNet-50 as a Backbone Model?

The question arises as to which CNN model is suitable as the backbone model for the system. We performed experiments with four state-of-the-art CNN models on the CBIS-DDSM dataset; the results are shown in Table 4. The results revealed that ResNet-50 outperforms the other models. The difference in the performance of the examined CNN models is attributed to the difference in their design strategies. In view of this, we employed ResNet-50.

### 4.2. The Effect of Multi-Scale and Multi-Context Schemes for a Test Region

To incorporate diverse context information in the system’s decision-making process, we introduced two schemes: multi-scale and multi-context ROIs, in Section 2.1. We performed experiments on the CBIS-DDSM dataset to see which is better. In these experiments, we used the whole training data for fine-tuning the backbone model, and the whole test data was used for evaluation. Also, we employed ResNet-50 as a backbone model and SVM with the polynomial kernel as a fusion method.

For the multi-scale scheme, we performed experiments with a single ROI and three different options, of multi-scale ROIs; the results are shown in Table 5. Among different multi-scale choices, {50,60,70,80,100} is the best choice; it gave an accuracy of 88.54%, which is approximately 15% higher than that of a single ROI. Similarly, the sensitivity, specificity, kappa, and F1 scores are significantly higher than those of a single ROI.

For the multi-context scheme, we considered three different choices, as shown in Table 6. The results in Table 5 show that “256 with five contexts” is the best choice; it achieved the best accuracy of 91.08%, a sensitivity of 87.12%, a specificity of 93.96%, kappa of 81.60%, and F1 score of 89.15%, are way better than those of single ROI. The multi-context choice “256 with 5 contexts” is the best of all; in onward experiments, we will use it. A comparison indicates that a multi-context scheme is better than a multi-scale scheme. It is probably due to the reason that the multi-context scheme encodes more diverse contextual information whereas the multi-scale scheme focuses more on scales. As in both cases, the performance is much better than a single ROI, it validates our hypothesis which is the basis for the design of the system.

### 4.3. The Effect of Different Fusion Techniques

To assess the impact of various fusion techniques described in Section 2.6, we performed experiments using the complete mass test dataset of CBIS-DDSM for evaluation with ResNet-50, fine-tuned using the complete mass training dataset of CBIS-DDSM, and the multi-context choice “256 with five contexts”. The results are presented in Table 7. The overall best technique is SVM with RBF kernel, which has an accuracy of 92.36%, a sensitivity of 99.24%, a specificity of 87.36%, Kappa of 84.70%, and F1-score of 91.61%. Although SVM with polynomial kernel and random forest give better specificities, their sensitivities are low; and sensitivity is more important. The results indicate that the stacking technique is better than other fusion techniques. This is due to the reason that stacking associates the weights to the predictions of multi-context ROIs according to their importance, learned from the data, whereas other techniques assign equal weights (i.e., 1) to the predictions.

### 4.4. The Effect of Density-Specific Models

We adapted ResNet-50 to make it density specific; the detail is given in Section 2.5. Each density-specific model was fine-tuned with the training set of the same density on the CBIS-DDSM dataset. We performed experiments using the density-specific models as backbone models in two different scenarios: Sc1—a density-specific model was tested on the test set of the same density (notation D*RresNet-50_D*_Ts_ in Table 8), Sc2—a density specific model was tested on the entire test set (notation D*ResNet-50 _D_Ts_ in Table 9). In all these cases, we used stacking (SVM with RBF kernel) as the fusion technique. Table 8 shows the comparative results on the density-specific models with ResNet-50. It can be observed that the density-specific models achieved overall higher performance than the original fine-tuned ResNet-50 model in both scenarios.

**Figure 14:**
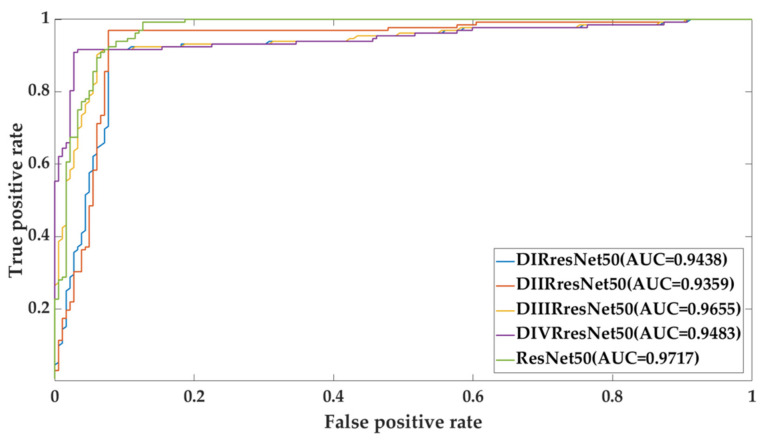
CBIS-DDSM ROC curve for the effect of density-specific models and ResNet50.

**Figure 15:**
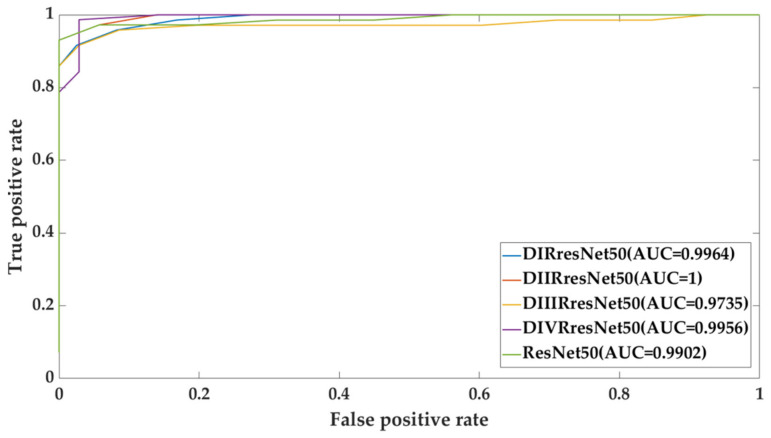
INbreast ROC curve for the effect of density-specific models and ResNet50.

Table 9 shows the results of the density-specific models for scenario Sc2 and the ResNet-50. Overall, the density-specific models yield better performance. In the CBIS-DDSM dataset, the density-specific model DIRresNet-50 gives the highest performance in terms of accuracy, kappa, and F1-score; it stands second in terms of sensitivity and specificity with a sensitivity of 98.4% and specificity of 92.31%. It indicates the model specific to density BI-RADS.I results in the overall best performance because the breast with BI-RADS.I is fatty, and patterns to discriminate benign and malignant masses are more apparent. The model learns these patterns and gains the potential to discern them in even dense breast areas. In the case of model DIVRresNet-50, sensitivity is very low; this is probably because in this case (BI-RADS.IV) the breast is dense, and it is difficult to discern the patterns of malignant masses. In the INbreast dataset, the density-specific model DIIRresNet-50 gives the highest performance in terms of accuracy, kappa, and F1-score; it stands first in terms of sensitivity and specificity with a sensitivity of 100% and specificity of 100%. Figure 14 and Figure 15 show the receiver operating characteristic (ROC) curve with the area under ROC (AUC) of all models, as presented in Table 10.

### 4.5. Comparison with State-of-the-Art Methods

For comparison, we selected similar state-of-the-art methods, which strictly followed the evaluation protocol of the CBIS-DDSM dataset. Table 10 summarizes the comparative results; the proposed method significantly outperforms the state-of-the-art methods. We selected the methods by Khan et al. [8] and Tsochatzidis et al. [10] for comparison on the CBIS-DDSM and those by Chougrad et al. [14], Ghada et al. [17], and Lou et al. [18] on the INbreast because they also employed ResNet-50. The performance of the proposed method is much better because we used the idea of the fusion of multi-context information extracted using ResNet-50 or its density-specific adaptations as backbone models. In addition, we used a novel augmentation approach for increasing the amount of data for fine-tuning the backbone models. Our proposed method achieved the highest accuracy of 94.90%, approximately 14% higher than the best method, on the CBIS-DDSM and 100%, approximately 5% higher than the best method. The method’s sensitivity is 99.24%, 100% on the CBIS-DDSM and INbreast respectively when ResNet-50 is used as a backbone model; it is much higher than those of the existing methods. The proposed method gives the overall best performance when density-specific model DIRresNet-50 and DIIRresNet-50 are used as a backbone model on the CBIS-DDSM and INbreast respectively.

## 5. Discussion

Leveraging the multi-context information, we developed a system to classify whether a breast mass region is benign or malignant and evaluated it on a benchmark dataset CBIS-DDSM and INbreast. A CNN model is used as a backbone model in the system; we evaluated some well-known CNN models (ResNet-50, DensNet-201, InceptionResNetV2, NasNetLarge) and found that ResNet-50 is the most appropriate model for the system. An important decision is about extracting contextual information; we evaluated two methods (multi-scale and multi-context ROIs) and found that multi-context ROIs represent the diverse contextual information in a better way and helps in better discrimination of benign and malignant mass regions. Multi-scale ROIs contain redundancy and include less diversity because a small ROI is fully contained in all larger scale ROIs, and due to this the performance of the system is not better than when multi-context ROIs are used. The multi-context ROI scheme MC2 (256 with 5 contexts) results in the best performance.

Another important factor that affects the performance of the system is the fusion technique. We applied different voting techniques (the majority of the decisions, soft voting, max voting) and stacking. The stacking yields better performance than the voting techniques. This is because the voting techniques give the same importance to all ROIs. In contrast, stacking assigns weights to the ROIs based on their contribution in discriminating benign and malignant masses; the weights are learned from the training data. We tried different base classifiers for stacking and found that SVM with RBF kernel is the best choice. The reason that SVM gives better performance is that it is based on the large margin theory.

Breast density is an important clinical feature used for assessing the risk for breast cancer. We made density-specific modifications (DIResNet-50, DIIResNet-50, DIIIResNet-50, DIVResNet-50) in ResNet-50 based on the idea of fusing local and global features and found that the system gives overall better performance on the CBIS-DDSM dataset (sensitivity of 98.48%, specificity of 92.31%, AUC of 94.38%, accuracy of 94.90%) when the density-specific model DIResNet-50 is used as a backbone model. Moreover, in the INbreast dataset (sensitivity of 100%, specificity of 100%, AUC of 100%, accuracy of 100%) when the density-specific model DIIResNet-50 is used as a backbone model. Furthermore, when the density-specific models are trained and tested on the same density type datasets, they classify the benign and malignant masses from the same breast density type in a better way. Also, their performance is better than ResNet-50, fine-tuned, and tested on density-specific datasets.

We compared the system with the state-of-the-art works that used the same CBIS-DDSM database split protocol. Khan et al. [8] employed the ResNet-50 model to extract features from a single view and then fused multi-view features. In [10], the authors tested the ResNet-50 model with two training scenarios. However, integrating different predictions of diverse multi-context ROIs from a single CNN model yields better classification results than using multiple CNN models with a single ROI, as in [9,11,12,13] also, as in [14,15,16,17,18] on the INbreast. This observation supports our hypothesis that different contexts surrounding the same mass object introduce diversity and lead to a better understanding of the nature of a mass. The key benefit of our method is robustness. It is computationally effective and needs less storage space.

**Figure 16:**
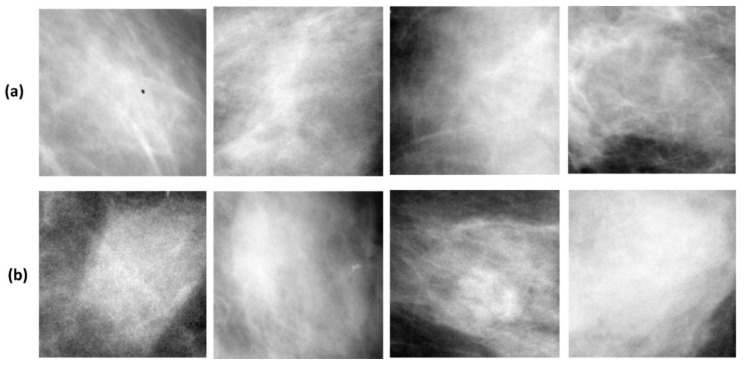
Example of mass regions, which are in dense surroundings, and it is difficult for the models to predict them correctly; (**a**) benign mass that is misclassified as a malignant mass; (**b**) malignant mass that is misclassified as a benign mass.

As with the majority of studies, the design of the current study is subject to limitations:

- The system uses ResNet-50 and its modified versions as the backbone model. It would be better if a new data-dependent model is designed which is adaptive to mammogram images.
- The method fails when the mass appears in extremely dense breast tissue because the characteristic similarity between the dense tissue and masses makes breast mass classification difficult. Figure 16 shows mass regions of test images that are difficult to classify accurately.

## 6. Conclusions

We addressed the challenging problem of discriminating benign and malignant masses and, leveraging the advances in deep learning, introduced a computer-aided system for this problem based on the hypothesis that multi-context information helps better differentiate benign and malignant masses. We evaluated the system thoroughly using the benchmark CBIS-DDSM and INbreast dataset and found that the system outperforms the state-of-the-art methods. For modeling the diverse contextual information, multi-context ROIs is the best scheme. For fusing the multi-context information extracted from multi-context ROIs, stacking is the best approach, and in stacking, SVM with RBF kernel serves as the best base model. Furthermore, ResNet-50 is the best backbone model for the system, and its density-specific modifications are even better than this model. Moreover, when ResNet-50 is adapted for a specific breast density type and fine-tuned on the dataset of the same density type, its density-specific modifications result in better discrimination of benign and malignant masses from the same breast density type. In the discrimination of benign and malignant masses, micro-texture patterns and the shapes of the mass regions play a key role. The proposed system implicitly exploits this information; an interesting future work will be to explicitly employ this information and adversarial learning to develop a more robust system.

## Figures and Tables

**Table 1:** ResNet-50 architecture for ImageNet.

Group	OUTPUT SIZE	50-Layer
$G^{1}$	112 × 112	7 × 7, 64, stride 2
$G^{2}$	56 × 56	3 × 3 max pool, stride 2
		$\left[\begin{array}{c} 1\times 1,\;64\\ 3\times 3,\;64\\ 1\times 1,\;256 \end{array}\right]\times 3$
$G^{3}$	28 × 28	$\left[\begin{array}{c} 1\times 1,\;128\\ 3\times 3,\;128\\ 1\times 1,\;512 \end{array}\right]\times 4$
$G^{4}$	14 × 14	$\left[\begin{array}{c} 1\times 1,256\\ 3\times 3,\;256\\ 1\times 1,1024 \end{array}\right]\times 6$
$G^{5}$	7 × 7	$\left[\begin{array}{c} 1\times 1,\;512\\ 3\times 3,512\\ 1\times 1,\;2048 \end{array}\right]\times 3$
	1 × 1	average pool, 1000-d fc, SoftMax

**Table 2:** Number of Curated Breast Imaging Subset of the Digital Database for Screening Mammography (CBIS-DDSM) mass ROI images.

Dataset	Dataset Set Pathology	Train	Test
D1 (BI-RADS.I)	Benign	103	24
	Malignant	128	21
D2 (BI-RADS.II)	Benign	257	74
	Malignant	248	70
D3 (BI-RADS.III)	Benign	145	63
	Malignant	125	29
D4 (BI-RADS.IV)	Benign	53	21
	Malignant	38	12
D (D1+D2+D3+D4)	Benign	558	182
	Malignant	539	132

**Table 3:** Number of INbreast mass ROI images.

Dataset	Dataset Set Pathology	Number of Masses
D1 (BI-RADS.I)	Benign	12
	Malignant	30
D2 (BI-RADS.II)	Benign	6
	Malignant	32
D3 (BI-RADS.III)	Benign	13
	Malignant	8
D4 (BI-RADS.IV)	Benign	6
	Malignant	1
D (D1+D2+D3+D4)	Benign	37
	Malignant	71

**Table 4:** The performance comparison on the CBIS-DDSM dataset using different convolutional neural networks (CNN).

Model	Sen (%)	SP (%)	ACC (%)	Kappa (%)	F1-Score
ResNet-50 [32]	99.24	87.36	92.36	84.70	91.61
DensNet-201 [52]	81.01	97.44	89.17	78.40	88.28
InceptionResNetV2 [53]	81.53	97.45	89.49	79	88.58
NasNetLarge [54]	81.25	98.70	89.81	79.70	89.04

**Table 5:** The effect of multi-scale ROIs on the CBIS-DDSM test performance.

Scheme	Sen (%)	SP (%)	ACC (%)	Kappa (%)	F1-Score
single	67.42	78.02	73.57	45.60	68.20
{5,10,15,20,25}-MS1	81.06	91.76	87.26	73.60	84.25
{10,20,30,40,50}-MS2	82.58	90.66	87.26	73.70	84.50
{50,60,70,80,100}-MS3	83.33	92.31	88.54	76.30	85.94

**Table 6:** The effect of the multi-context of the CBIS-DDSM test ROI.

Scheme	Sen (%)	SP (%)	ACC (%)	Kappa (%)	F1-Score
single	67.42	78.02	73.57	45.60	68.20
256 with 3 contexts-MC1	73.48	86.26	80.89	60.40	76.38
256 with 5 contexts-MC2	87.12	93.96	91.08	81.60	89.15

**Table 7:** The effect of different fusion techniques on the CBIS-DDSM dataset.

Fusion Technique		Sen (%)	SP (%)	Acc (%)	Kappa (%)	F1-Score
**Stacking**	Random Forest	87.88	93.96	91.40	82.30	89.59
	SVM with RBF	99.24	87.36	92.36	84.70	91.61
	SVM with Linear	92.42	92.31	92.36	84.40	91.04
	SVM with Polynomial	87.12	93.96	91.08	81.60	89.15
Majority voting		77.27	83.52	80.89	60.80	77.27
Soft Voting		78.79	84.62	82.17	63.10	78.79
Max voting		81.06	84.07	82.80	64.40	79.85

**Table 8:** The comparison between density-specific models ResNet-50; D*RresNet-50_D*_Ts_ means the ResNet-50 model adapted for density type “*”, fine-tuned with the training CBIS-DDSM dataset D*_T_ and was tested on test dataset D*_TS_.

Models	Sen (%)	SP (%)	ACC (%)	Kappa (%)	F1-Score
DIRresNet-50_D1_T__D1_Ts_	100	91.67	95.56	91.12	95.45
DII RresNet-50_D2_T__D2_Ts_	92.86	87.84	90.28	80.57	90.28
DIII RresNet-50D3_T__D3_Ts_	96.55	96.83	96.74	92.50	94.92
DIVRresNet-50_D4_T__D4_Ts_	91.67	100	96.97	93.30	95.65
ResNet-50_D1_T_ _D1_Ts_	90.48	87.50	88.89	77.70	88.37
ResNet-50_D2_T_ _D2_Ts_	90	75.68	82.64	65.40	83.44
ResNet-50_D3_T_ _D3_Ts_	82.76	84.13	83.70	63.90	76.19
ResNet-50_D4_T_ _ D4_Ts_	66.67	100	87.88	71.80	80

**Table 9:** The effect of density specific models, D*RresNet-50_D*_Ts_ means the ResNet-50 model adapted for density type “*”, Fine-tuned with the training dataset D*_T_ and was tested on test dataset D*_TS_.

Datasets	Models	Sen (%)	SP (%)	ACC (%)	Kappa (%)	F1-Score
CBIS-DDSM	DIRresNet-50_ D_T__D_Ts_	98.48	92.31	94.90	89.65	94.20
	DIIRresNet-50_D_T__D_Ts_	96.97	92.31	94.27	88.36	93.43
	DIIIRresNet-50_D_T__D_Ts_	97.73	91.21	93.95	87.75	93.14
	DIVRresNet-50_D_T__D_Ts_	91.67	96.70	94.59	88.83	93.44
	RresNet-50_D_T__ D_Ts_	99.24	87.36	92.36	84.67	91.61
INbreast	DIRresNet-50_ D_T__D_Ts_	100	97.5	99.09	98.07	99.31
	DIIRresNet-50_D_T__D_Ts_	100	100	100	100	100
	DIIIRresNet-50_D_T__D_Ts_	97.14	97.5	97.19	94.31	97.77
	DIVRresNet-50_D_T__D_Ts_	100	97.14	99	97.73	99.26
	RresNet-50_D_T__ D_Ts_	98.57	97.5	98.18	96.14	98.61

**Table 10:** The comparison with different state-of-the-art methods.

	References	Models\Descriptors	Sen (%)	SP (%)	AUC (%)	ACC (%)
CBIS-DDSM	Khan et el. [8]	ResNet-50	75.46	62.75	69.10	69.98
		MVFF	81.82	72.02	76.90	77.66
	Tsochatzidis et al. [10]	ResNet-50 from scratch	-	-	80.40	74.90
		Fine-tuning ResNet-50	-	-	63.70	62.70
	Duggento et al. [11]	AlexNet	84.40	62.44	-	71.19
	AL Hakeem and Jang [13]	LBP-HOG	-	-	-	64.35
	Li et al. [9]	Dual-core Net	-	-	85	-
	Shu et al. [12]	Region-based Group-max Pooling	-	-	83.3	76.2
		Global Group-max Pooling	-	-	82.3	76.7
	Proposed system	Multi-context ResNet-50	99.24	87.36	97.17	92.36
		Multi-context DIRresNet-50	98.48	92.31	94.38	94.90
		Multi-context DIIRresNet-50	96.97	92.31	93.59	94.27
		Multi-context DIIIRresNet-50	97.73	91.21	96.55	93.95
		Multi-context DIVRresNet-50	91.67	96.70	94.83	94.59
INbreast	Chougrad et al. [14]	Resnet-50	-	-	-	92.50
	Al-antari et al. [15]	Alex Net	97.14	92.41	94.78	95.64
	Shen et al. [16]	ResCU-Net	-	-	96.16	94.12
	Ghada et al. [17]	ResNet	-	-	-	90
		Inception	-	-	-	95
	Lou et al. [18]	ResNet-50	69.23	74	84.96	72.37
		MGBN-50	77.16	88.24	93.11	84.50
	Proposed system	Multi-context ResNet-50	98.57	97.500	99.02	98.18
		Multi-context DIRresNet-50	100	97.5	99.64	99.09
		Multi-context DIIRresNet-50	100	100	100	100
		Multi-context DIIIRresNet-50	97.14	97.50	97.35	97.19
		Multi-context DIVRresNet-50	100	97.14	99.56	99

## Data Availability

The CBIS-DDSM dataset is available at: https://wiki.cancerimagingarchive.net/display/Public/CBIS-DDSM (accessed on 5 October 2021).
